# Understanding the effects of transcranial direct current stimulation on the neurovascular unit: a narrative review

**DOI:** 10.3389/fnins.2025.1667100

**Published:** 2025-10-01

**Authors:** Aidan Lewis, Ben Rattray, Andrew Flood

**Affiliations:** ^1^Discipline of Psychology, Faculty of Health, University of Canberra, Canberra, ACT, Australia; ^2^Institute of Sport Science, Human Movement Science, University of Bundeswehr Munich, Neubiberg, Germany; ^3^Discipline of Exercise Science, Faculty of Health, University of Canberra, Canberra, ACT, Australia; ^4^Research Institute for Sport and Exercise, University of Canberra, Canberra, ACT, Australia

**Keywords:** tDCS, mechanisms, neuromodulation, review, neurovascular unit

## Abstract

Transcranial direct current stimulation (tDCS) is a non-invasive neuromodulation technique that has demonstrated promise both for treating diverse clinical conditions and for enhancing brain function in healthy adults. Despite increasing popularity, the precise physiological mechanisms underlying its effects remain poorly defined, contributing to inconsistent findings. This review synthesises current evidence for both acute and enduring effects of tDCS across the complete neurovascular unit, encompassing neurons, astrocytes, oligodendrocytes, microglia, the blood–brain barrier, metabolic pathways, and immune responses. We review recent transcriptomic, proteomic, and metabolomic studies which reveal that tDCS induces coordinated molecular changes, including modulation of genes involved in inflammation, neurogenesis, calcium signalling, mitochondrial metabolism, and synaptic plasticity proteins. We emphasise significant gaps in current literature, particularly the limited consideration given to astrocytes and oligodendrocytes, despite their known importance in activity-dependent plasticity. We conclude that the neurovascular unit represents an integrative and complex target of tDCS, suggesting that comprehensive modulation of these components may better explain observed changes in cognitive, behavioural, and neuropsychological outcomes. Future research should move beyond a neuron-centric perspective, embracing a more integrative framework that considers interactions amongst all elements of the neurovascular unit. Such a holistic approach will enhance our understanding of how tDCS exerts its effects, thereby improving its clinical utility.

## 1 Introduction

Transcranial direct current stimulation (tDCS) is a non-invasive brain stimulation technique that delivers a weak electrical current to the brain, modulating the function of brain cells and influencing behavioural outcomes (Nitsche and Paulus, 2000). tDCS has emerged as a tool for both enhancing human capabilities and treating various neurological disorders (Nitsche and Fregni, 2007; Pustovrh, 2014). Due to its affordability, ease of application, minimal side effects, and broad potential applications, the technique has accumulated significant interest from both popular media (McCarthy-Jones, 2017; Ruddick, 2019), and the research community (Day et al., 2022; Riggall et al., 2015). For example, over 8,000 peer-reviewed documents relating to tDCS have been published since 2000, with more than 4,000 of these articles published since 2020 (PubMed search, “transcranial direct current stimulation”). This “explosion” of research reflects the scientific community’s interest in its prospective benefits, resulting in a substantial body of literature exploring its uses.

Despite the excitement and promise surrounding tDCS, criticism regarding its validity and utility persists. Several scholarly critiques (Bestmann et al., 2015; Horvath et al., 2015), and meta-analyses suggest that tDCS has no reliable effects on behaviour (Müller et al., 2022; Saleh et al., 2023) due to irreproducible effects (Horvath et al., 2014) and heterogenous findings (Klees-Themens and Théoret, 2023). Indeed, the inconsistency and variability of tDCS limits its clinical application, with the technique currently approved by the Australian Therapeutic Goods Administration and European Union Medical Device Regulation *only* for unipolar depression, major depressive disorder, and chronic pain (Royal Australian and New Zealand College of Psychiatrists, 2022), despite a plethora of tDCS research in other psychological conditions including anxiety (Garcia et al., 2020), schizophrenia (Brunelin et al., 2012), attention deficit hyperactive disorder (Salehinejad et al., 2017), and autism (García-González et al., 2021). Contributing to the uncertainty, the mechanisms by which tDCS affects brain function remain poorly understood. There is limited understanding of how tDCS impacts neural functions at the level of individual neurons and local microcircuits, particularly in humans (Parkin et al., 2019). Arguments suggest that these knowledge gaps hinder hypothesis-driven research and the interpretation of findings: *“The use of* [tDCS] *has thus outpaced the mechanistic rationales for its application. This is no trivial matter, because these gaps in our knowledge delay the development of more effective and ever-safer stimulation protocols, lead to wastefulness when applications are based on spurious rationales, and promote the proliferation of implausible mechanistic inferences”* (Bestmann et al., 2015).

Despite two decades of intensive investigation, the physiological mechanisms of tDCS remain incompletely mapped, and the technique continues to yield heterogeneous results within and between participants (Chew et al., 2015). Closing this mechanistic knowledge-gap is now a critical pre-requisite for sound dose design, reproducible experimental work, and clinical translation. Earlier syntheses have advanced the field but left key questions open. Pelletier and Cicchetti (2015) mapped the intracellular pathways governing inflammation and neurogenesis after direct current stimulation, whereas Bikson et al. (2019) and Stagg et al. (2018) focused on shifts in neuronal excitability and network physiology. More recently, Bahr-Hosseini and Bikson (2021) broadened the lens by charting tDCS-evoked vascular responses along the arterial tree. Even so, existing syntheses remain predominantly neuron-centric, offering only cursory attention of the wider neurovascular unit—astrocytes, oligodendrocytes, pericytes, microglia, and the blood–brain barrier—as well as the emerging roles of cerebrospinal-fluid and glymphatic exchange.

**Figure 1:**
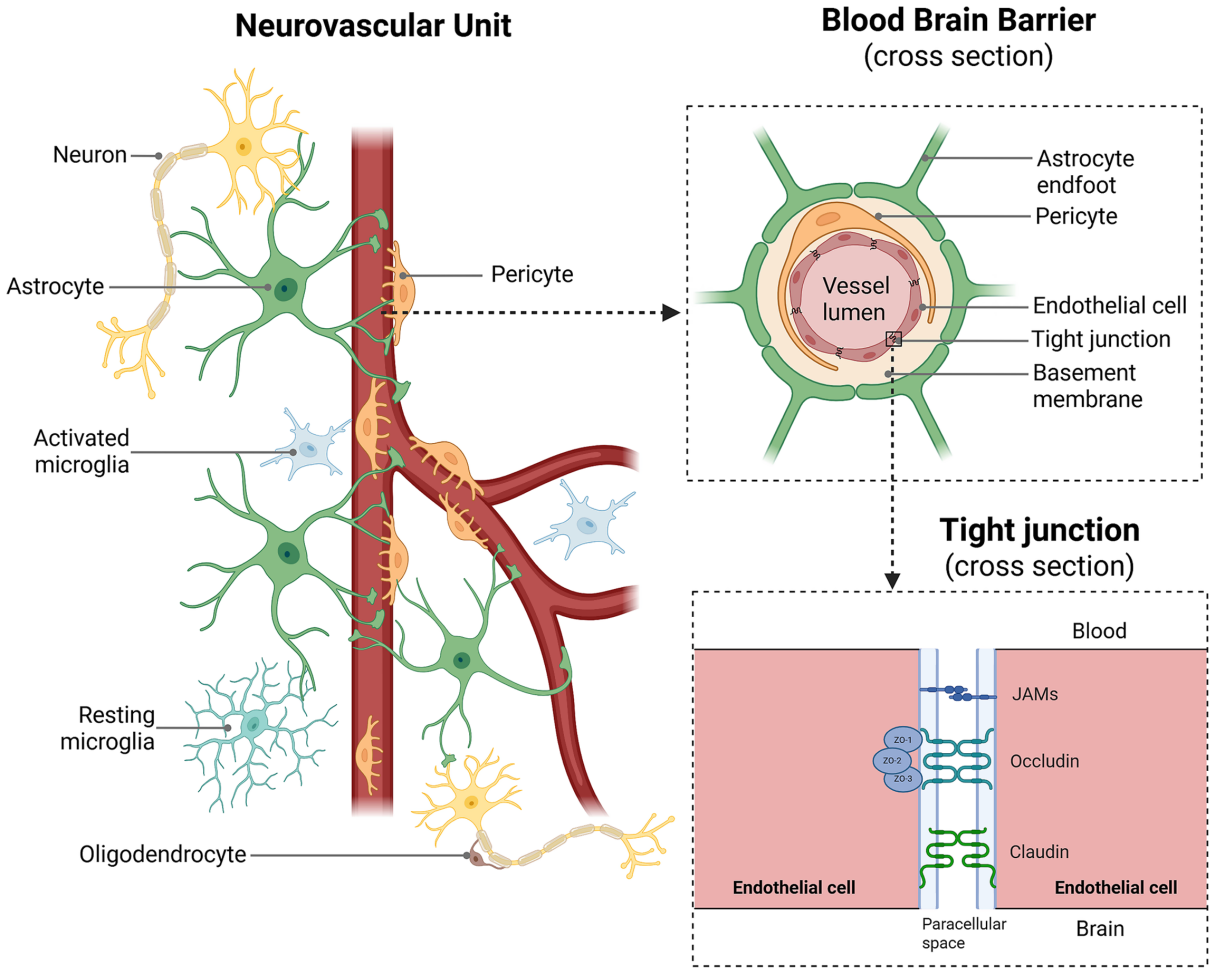
A visual depiction of the elements of the neurovascular unit. The neurovascular unit, blood brain barrier and its tight junctions have all been proposed to be impacted by tDCS. Created in BioRender. Lewis (2025) https://BioRender.com/4rg7khk.

Due to the heterogeneity of the literature that spans multiple species, models, and temporalities, we provide a narrative review to address the aforementioned shortfall. Here, the aim is to provide a deeper understanding of how tDCS impacts each element of the neurovascular unit and its implications for clinical translation. Drawing on the latest molecular, cellular, haemodynamic and immuno-metabolomic evidence, we present an integrated view of tDCS effects, spanning immediate effects on single cells to enduring adaptations across neuronal networks. We first discuss the immediate effects of stimulation, showing how electric-field orientation, montage geometry and tissue conductivity jointly impact responses in both neuronal and non-neuronal elements. We then discuss the enduring effects of stimulation that persist upon stimulation cessation, highlighting the drivers and modulators of plasticity, as well as emerging contributors of the neurovascular unit including astrocytes, microglia, and oligodendrocytes, all of which may explain the technique’s variable efficacy. It should be noted that the relative length of each section mirrors the current evidence base: the literature is dominated by studies on neuronal effects, with fewer investigations into the wider neurovascular unit. Figure 1 provides an overview of the neurovascular elements discussed in this review.

Building on earlier syntheses, we incorporate the most recent evidence on elements of the neurovascular unit that have been largely overlooked. We extend and synthesise findings on how tDCS modulates gene expression, aiming to holistically explain how tDCS may exert its behavioural and cognitive effects. Our aim is to provide researchers with a comprehensive review of all known mechanisms of action of tDCS that spans molecular and cellular processes through to whole-brain dynamics. To respect the distinct timescales of tDCS action, we begin with its acute neuronal effects at multiple organisational levels, proceed to longer-lasting neuronal adaptations, before examining the non-neuronal effects of tDCS and how these may address the heterogeneity seen in the literature.

## 2 The neuronal mechanisms underlying tDCS

Observable changes in psychophysiological outcomes during or following tDCS administration are largely attributed to the effects of tDCS on neurons (Nitsche and Paulus, 2000). Neurons are electrically excitable cells, and their function depends critically on the generation of action potentials, which are elicited when the resting membrane reaches a certain potential threshold (usually −50 mV). The application of tDCS initiates the polarisation of the cell membranes (Bikson et al., 2004), but this change in cell membrane potential is subthreshold and does not elicit action potentials themselves. Indeed, tDCS delivers a current intensity between 1 and 2 mA which is too low to directly elicit action potentials (the peak current density at any point in the brain is roughly 0.05–0.5 A/m^2^). Despite being subthreshold, the polarisation of cell membranes has multifaceted consequences that extend beyond the resting potential of the cell itself, impacting various aspects of neuronal function, including action potential threshold and timing, whole brain oscillations, synaptic plasticity, and morphological and molecular changes (Bikson et al., 2004; Bindman et al., 1964; Marshall et al., 2016; Radman et al., 2009; Reato et al., 2014). These diverse neurophysiological effects can be viewed as being secondary to the primary consequences of tDCS for the polarisation of cell membranes. These neurophysiological effects will be discussed alongside their relationship to the behavioural, cognitive, and neuropsychological changes observed following tDCS administration.

### 2.1 Acute effects of tDCS on neurons

#### 2.1.1 Membrane polarisation

tDCS stimulation produces electrical current flow across the brain (Datta et al., 2009; Huang et al., 2017; Miranda et al., 2006; Opitz et al., 2016), with current from the anode flowing into the brain and then exiting at the site of the cathode. This flow of current around neurons results in the polarisation of cell membranes. Flow into a specific membrane compartment (from outside the neuron into it) will result in local membrane hyperpolarisation, and flow out of another membrane compartment (from inside to out) will result in local membrane depolarization (Bikson et al., 2004; Andreasen and Nedergaard, 1996). At the single neuron level, the physics of electrical stimulation dictate that any neuron exposed to extracellular direct current (DC) stimulation will have some compartments that are depolarized while others are hyperpolarized (Bikson et al., 2004; Chan et al., 1988). Which compartments are polarised, and in which direction, depends on the neuronal morphology relative to the DC electric field. For a typical cortical pyramidal neuron, with a large apical dendrite pointed towards the cortical surface, a surface anode (positive electrode, generating a cortical inward current flow) will result in somatic depolarisation and apical dendrite hyperpolarisation (Radman et al., 2009). For this same neuron, a surface cathode (negative electrode, generating cortical outward current flow) will result in somatic hyperpolarisation and apical dendrite depolarisation. This pattern has motivated a term called the “somatic doctrine,” which holds that tDCS-evoked shifts in excitability arise chiefly from changes in somatic membrane potential (Bikson et al., 2019).

The soma is indeed central to action-potential initiation; yet mounting evidence indicates that the decision to fire also reflects the integrated activity of the entire neuronal architecture, including dendrites, axon, presynaptic terminals, and axon hillock (Bikson et al., 2004; Bindman et al., 1964; Radman et al., 2009; Purpura and McMurtry, 1965). Direct-current fields polarise these other compartments as well: afferent axons and their terminals experience morphology-dependent shifts in membrane potential during DC stimulation, sometimes with polarity opposite to that of the soma, thereby shaping synaptic transmission (Kabakov et al., 2012; Márquez-Ruiz et al., 2012; Ranck, 1975; Tranchina and Nicholson, 1986). DC fields can modulate the functionality of all these compartments, increasing the complexity of a purely “somatic doctrine” (Kabakov et al., 2012; Kronberg et al., 2017; Rahman et al., 2013). Despite the involvement of all compartments of the neuron, the somatic doctrine has predominantly and implicitly informed the rationale for most tDCS human trials – namely presumed excitation by the anode and inhibition by the cathode.

While the respective effects of anodal and cathodal tDCS on the soma have been extended to the whole neuron, they cannot generalise to the effects on the excitability of the whole brain. The neuronal morphology inside the brain is heterogenous. Current that is passed through tDCS electrodes takes a path through the head determined by individual anatomy and the resistivity of each tissue type (Neuling et al., 2012). A fraction of the current never crosses the resistive cranium, instead shunting across the relativity conducive (low resistivity) scalp (Paulus and Rothwell, 2016). Of the current fraction that crosses the skull, a further portion is shunted by the high conductivity cerebrospinal fluid (Neuling et al., 2012). The current component that reaches the brain crosses the grey and then white matter (Lee et al., 2015). As current crosses brain tissue, it generates an electric field on the local tissue, which neurons are then exposed to. However, due to the lack of uniformity across other tissue types (i.e., skull thickness, skin conductance) between participants, a resultant heterogeneity in electric field intensity distribution across the cortex occurs (Mosayebi-Samani et al., 2021). Considering this, the targeted application of tDCS is challenging given the inconsistent electric field delivered to the brain across individuals.

**Figure 2:**
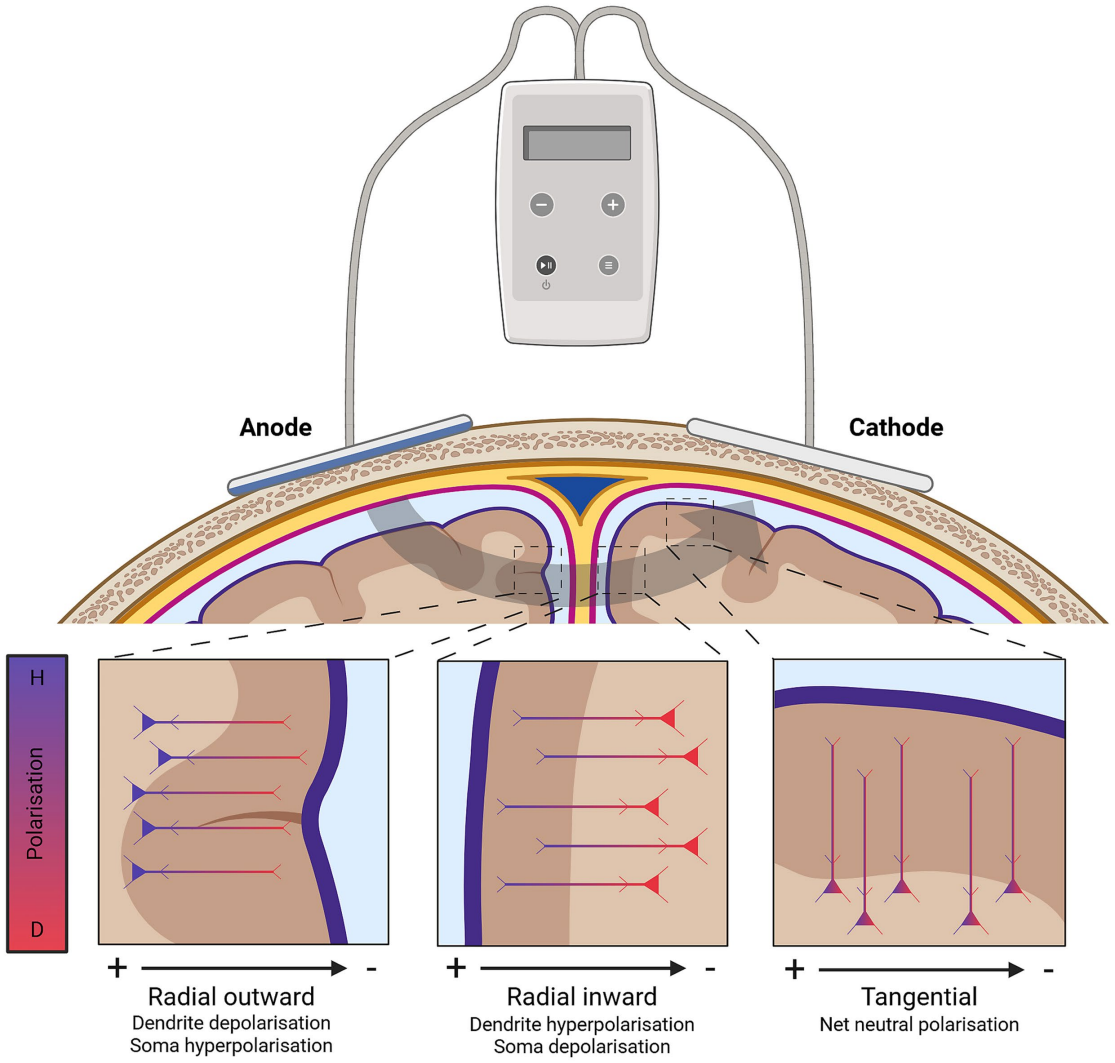
A visual representation of the impact of cortical folding and orientation of neurons on how tDCS-derived electric fields impact polarisation. Created in BioRender. Lewis (2025) https://BioRender.com/grakwub.

In addition to varied current intensity that crosses into the brain tissue, the direction of current flow resulting from tDCS is also varied. The direction of current flow across the grey matter can be radial inward (from the pial surface towards grey/white matter boundary), radial outward, or tangential (along the grey matter) (Rahman et al., 2013). The direction of current with respect to the orientation of the somato-dendritic axes of neurons being stimulated is a primary determinant of the physiological impact of tDCS (Bikson et al., 2004; Rahman et al., 2013; Farahani et al., 2021; Lafon et al., 2017; Rahman et al., 2015). Current flowing parallel to the somato-dendritic axis (radial orientation) can cause somatic depolarisation or hyperpolarisation: current flowing inward (radial inward) from dendrite to soma causes depolarisation, whereas current flowing outward (radial outward) from soma to dendrite causes hyperpolarisation. By contrast, current applied orthogonally to the somatodendritic axis – hereon referred to as “tangential” orientation (along the grey matter) – results in little to no somatic polarisation (Bikson et al., 2004; Rahman et al., 2013; Farahani et al., 2021; Lafon et al., 2017; Rahman et al., 2015). Although computational models often treat the electric field beneath each electrode as uniform in strength and orientation, individual differences in skull thickness, cortical folding, and other anatomical features generate appreciable field variability—even directly under the stimulating electrodes (Rahman et al., 2013; Lafon et al., 2017). These local disparities can drive neurons towards opposite polarisation states (depolarisation in some elements, hyperpolarisation in others; see Figure 2), perhaps explaining the diverse behavioural effects reported across studies. Such variability highlights the limits of one-compartment explanations and underscores the need for *in vivo* measures that capture the integrated response of entire pathways rather than isolated cells.

#### 2.1.2 Motor evoked potentials

One method for quantifying online, pathway-level excitability effects of tDCS is the motor-evoked potential (MEP). Nitsche and Paulus (2000) first paired 4 s of 1 mA anodal tDCS with single-pulse TMS and recorded an immediate (online) increase in twitch amplitude of the contralateral hand, consistent with a transient boost in the number or synchrony of descending corticospinal volleys (Bestmann et al., 2015; Nitsche et al., 2003a; Tremblay et al., 2016). Follow-up work on online MEPs is sparse: a handful of reports have replicated the facilitation and even linked its size to stronger after-effects measured minutes later (Nitsche et al., 2003a; Bergmann et al., 2009; Liebetanz et al., 2002), yet a near-replication by Pillen et al. (2022) did not detect any change in online MEP size. Conversely, Santarnecchi et al. (2014) found no immediate effect but did see a post-stimulation increase, suggesting that the principal action of tDCS might occur only once current flow ceases. Such discrepancies highlight the multifaceted nature of the measurement itself. An MEP reflects not only the excitability of cortical pyramidal neurons, but also the state of inter-neuronal circuits, spinal motoneurons, as well as peripheral axons—all of which can be differentially influenced by the weak, spatially heterogeneous fields produced in vivo. MEPs also ignore glial partners in the neurovascular unit; astrocytes, oligodendrocytes and microglia can alter ion homeostasis, neurotransmitter clearance and microvascular tone (De Luca et al., 2020), shifting neuronal thresholds or synchrony without necessarily enlarging the evoked potential. With little research focused on the during-stimulation window—and most of the literature targeting post-stimulation outcomes—it is unsurprising that results remain heterogeneous. This unclear evidence points to the need for alternative approaches to understanding tDCS effects, from single-neuron measures of firing rate and spike timing to population-level markers such as oscillatory dynamics and neurovascular signals, which may offer a clearer understanding of the mechanisms of action of tDCS.

#### 2.1.3 Action potential firing rate

Changes to individual neurons and whole brain excitability resulting from tDCS are important because they produce changes in action potential firing rate. Early animal studies using weak DCS showed a change in ongoing action potential discharge rate that shares a roughly linear relationship with electric field intensity (Bindman et al., 1964; Purpura and McMurtry, 1965; Creutzfeldt et al., 1962; Terzuolo and Bullock, 1956). Some of the earliest work by Terzuolo and Bullock (1956) reported a detectable change in neuronal firing rate at electric fields as small as 0.8 V/m, and Bindman et al. (1964) showed classic polarity-dependent effects—anodal currents raised, whereas cathodal currents lowered, cortical firing rates. Subsequent in-vivo recordings have refined that threshold. Tanaka et al. (2020) reported that *both* anodal and cathodal tDCS boosted single-unit firing beneath the electrode in anaesthetised rat cortex, while Farahani et al. (2024) demonstrated monotonic rate increases down to 0.35 V/m in mouse hippocampus – equivalent to 0.4 A/m^2^ current densities delivered in standard human protocols and far below the 34.2 A/m^2^ often used in earlier animal studies (Brunoni et al., 2011a). Together, these findings overturn the notion that only high-intensity laboratory fields influence neuronal output.

Increases in mean firing rate do not automatically translate into reliable behavioural or cognitive gains. Single neurons embedded in a complex circuit can polarise in opposite directions depending on cell type, morphology, and compartment, so population-level effects may cancel out. Further, tDCS alters much more than how often neurons fire; but also, when they fire, how they synchronise, and how synaptic inputs are weighted, all of which are factors that can dominate network function even when average firing rates shift only modestly. Indeed, Milighetti et al. (2020) caution that behavioural and cognitive effects reported in previous tDCS studies are likely driven by effects other than changes in spontaneous neuronal firing.

The evidence therefore points to a two-step conclusion: clinically relevant electric fields are indeed sufficient to modulate firing rate, but firing rate shifts alone are not enough to induce changes in behaviour. The next section builds on this by examining timing-based and network-level mechanisms that may better account for the variability of tDCS outcomes.

#### 2.1.4 Action potential timing

Weak electric fields generated by tDCS affect not only how often neurons fire (action potential firing rate) but also when they fire. Spike timing—the precise millisecond at which an action potential occurs relative to the neuron’s own membrane trajectory or to ongoing network rhythms—can change synaptic integration and plasticity even when the average firing rate remains constant. Experimental work on hippocampal neurons illustrates this: Radman et al. (2007) applied a spatially uniform field to rat primary motor cortex during steady depolarisation and found that anodal stimulation advanced the moment a neuron crossed threshold, whereas cathodal stimulation delayed it, thereby shifting spike timing without altering spike count. Similar timing shifts appear at the population level: in cortical slices, Reato et al. (2010) observed that depolarising fields compressed, and hyperpolarising fields stretched, the intervals between peaks in local-field potentials of rat hippocampal slices, indicating advances or delays across many cells. Computational models reproduce the same pattern, showing cathodal DCS systematically advances and anodal DCS delays spikes relative to unstimulated controls (Lafon et al., 2017).

These millisecond adjustments, however, do not automatically translate into long-lasting synaptic change. Plasticity emerges only when tDCS acts on circuits that are already active; a network “at rest” shows weak polarisation but no enduring synaptic modification (Márquez-Ruiz et al., 2012; Fritsch et al., 2010). This qualifier is critical for interpreting behavioural outcomes: while tDCS instantaneously polarises membranes and alters both firing rate and spike timing, the behavioural and cognitive gains reported in humans likely stem from activity-dependent plasticity and broader network reorganisation, not from single-neuron effects alone. Moreover, because current density and field orientation vary markedly across the folded human cortex, spike-timing shifts will differ from one region or cell type to another. Such heterogeneity challenges the convenient—but overly simplistic—rule that “anodal depolarises” and “cathodal hyperpolarises,” and underscores the need to examine how tDCS reshapes activity in larger neural and neurovascular networks.

#### 2.1.5 Oscillations

Neuronal oscillations emerge when large ensembles of neurons fire rhythmically; the frequency reflects how quickly successive population volleys occur, whereas the amplitude (power) scales with the proportion of cells that discharge in synchrony (Buzsáki and Draguhn, 2004). At the single-cell level, the firing-rate and spike-timing effects reviewed in Sections 2.1.2–2.1.3 form the foundation for these network rhythms: when many neurons shift their timing together, the power of the resulting population oscillation rises or falls. Oscillatory power therefore explains how micro-scale excitability changes lead to changes at the macro-scale network dynamics.

In humans, functional magnetic resonance imaging has shown that tDCS generally alters activity in regions near the anode, but also in distant brain structures that are functionally connected (Amadi et al., 2014; Hunter et al., 2015; Krishnamurthy et al., 2015). These findings indicate the possibility of tDCS to induce changes in large-scale oscillatory coupling. EEG and MEG studies have examined this possibility more directly, yet the pattern is inconsistent. For example, McDermott et al. (2019) reported that occipital anodal tDCS impaired visual working-memory accuracy, elevated resting theta power in prefrontal cortex, raised alpha power in occipital cortex, and suppressed task-related frontal theta—all effects that could reflect downstream propagation from the stimulated site. Over primary motor cortex, anodal stimulation has been found to boost alpha- and beta-band power during motor imagery and execution (Mondini et al., 2018; Wei et al., 2013), whereas cathodal currents reduce it (Baxter et al., 2017); however, several groups using comparable montages have observed no significant changes (Gordon et al., 2018; Luft et al., 2018). A recent meta-analysis pooling 39 experiments concluded that, on average, neither resting-state nor event-related oscillatory power differs reliably from sham stimulation (Chan et al., 2021). In short, tDCS can shift oscillatory dynamics, but the direction, size, and behavioural relevance of those shifts are highly context dependent. Moreover, since most behavioural improvements emerge after the current is switched off, we need to examine the stimulation’s lasting effects and the mechanisms by which its immediate actions consolidate into longer-term changes—topics addressed in the following section.

### 2.2 Enduring effects of tDCS on neurons

**Figure 3:**
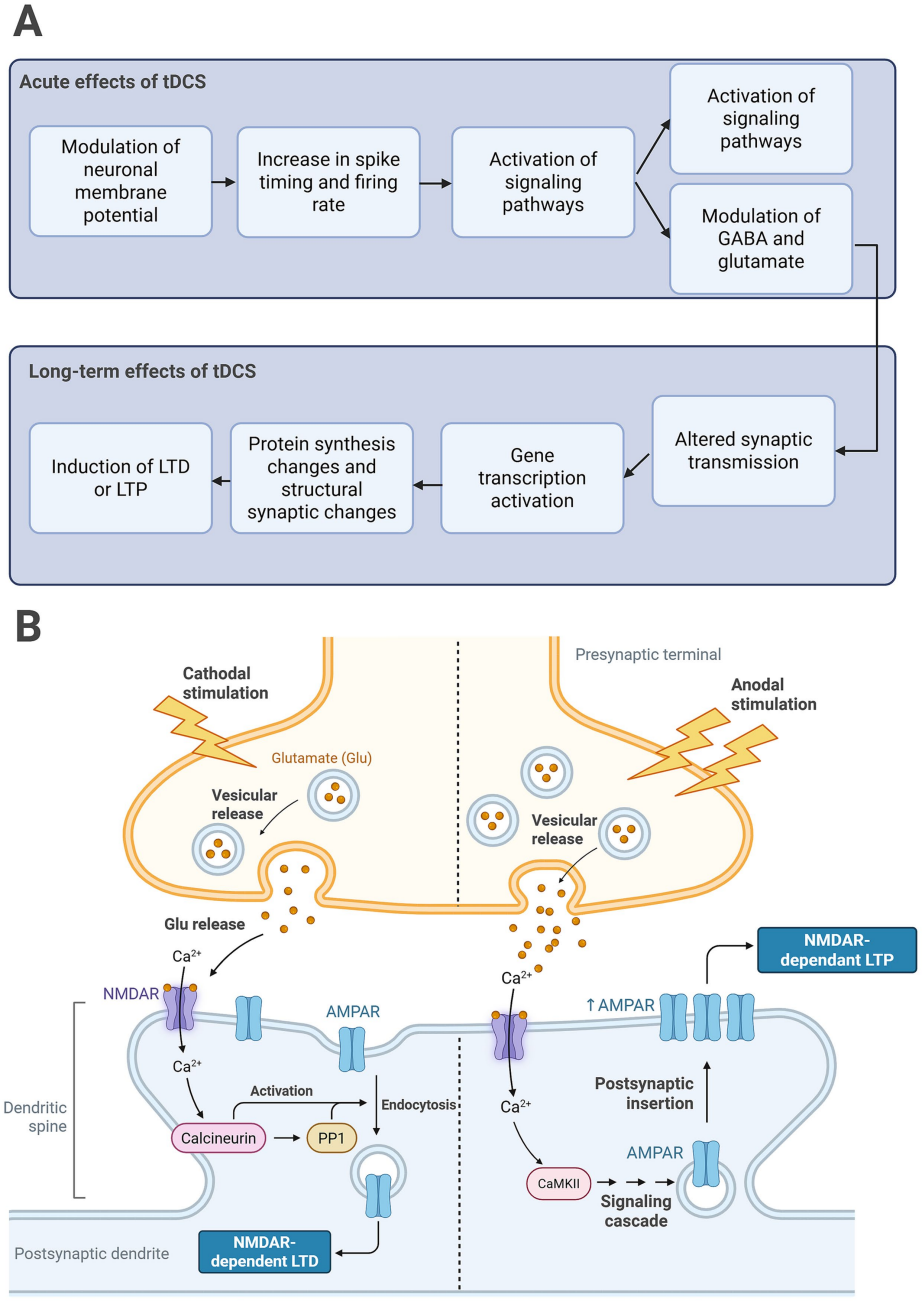
**(A)** The proposed pathway from acute tDCS effects into the enduring changes at the synapse. **(B)** A visual representation of how tDCS induce LTD and LTP-like effects. Created in BioRender. Lewis (2025) https://BioRender.com/2ve210s.

Beyond its immediate and online effects, tDCS can continue to shape neuronal excitability for minutes to hours after stimulation cessation (Kuo et al., 2013). In the human motor cortex, the polarity-dependent pattern seen during stimulation—anodal facilitation, cathodal suppression—often re-emerges immediately after current offset. For example, Nitsche and Paulus (2000) and Fricke et al. (2010) both recorded larger MEPs in the first seconds following anodal tDCS, an effect replicated in several subsequent studies (López-Alonso et al., 2015; Siebner et al., 2004). The duration of these after-effects scales with stimulation length. Stimulation between 5 and 7 min fade within ∼30 min, whereas ≥9 min of stimulation extend the effect to 60–90 min (Nitsche and Paulus, 2000). Longer follow-ups show still-prolonged excitability: Vaseghi et al. (2015) reported MEPs peaking ~30 min post-tDCS and remaining above baseline 24 h later; similar trajectories have been observed for oscillatory power (Romero Lauro et al., 2014) and for MEPs in other cohorts (Vaseghi et al., 2015; Jamil et al., 2017). These sustained changes are widely attributed to NMDA-dependent synaptic plasticity—long-term potentiation (LTP) and depression (LTD)—that alters the efficacy of neuronal firing (Malenka and Bear, 2004). LTP is a marked increase in synaptic strength that emerges when bursts of presynaptic spikes coincide with large and concurrent postsynaptic depolarisation (Hulme et al., 2014). This removes the Mg^2+^ block on NMDA receptors, allowing Ca^2+^ to flood the dendritic spine and activate kinase cascades that insert additional AMPA receptors and enlarge the spine head, thereby amplifying future transmissions (Kronberg et al., 2017). LTD, in contrast, is a sustained weakening that follows lower-frequency or poorly timed activity; the smaller Ca^2+^ rise favours phosphatase pathways that remove AMPA receptors and shrink the spine, diminishing synaptic efficacy (Lüscher and Malenka, 2012). Figure 3 illustrates this process. There are two main drivers of this neuronal plasticity (Arckens et al., 2000), as well as a range of modulators of neuronal plasticity (Pedrosa and Clopath, 2017), which may be targeted by tDCS. Understanding the underlying mechanisms of neuronal plasticity and how tDCS may impact its drivers and modulators is critical for understanding of how tDCS can influence behavioural outcomes.

#### 2.2.1 Drivers of plasticity

##### 2.2.1.1 Glutamate

NMDA-type glutamate receptors regulate the influx of calcium (Ca^2+^) ions in response to coincident pre- and postsynaptic activity, thereby determining whether synapses undergo LTP or LTD (Citri and Malenka, 2008). Pharmacological studies have clearly demonstrated that tDCS exerts its plasticity-modulating effects predominantly via these NMDA receptors. Specifically, administration of the NMDA receptor antagonist dextromethorphan in humans abolishes both LTP- and LTD-like effects typically induced by tDCS (Liebetanz et al., 2002; Nitsche et al., 2003b; Stefan et al., 2002; Wolters et al., 2003), whereas treatment with the partial NMDA receptor agonist d-cycloserine extends and enhances the LTP-like effects induced by anodal tDCS (Nitsche et al., 2004a). Recent experimental findings further highlight the pivotal role of glutamatergic NMDA receptor activity in tDCS-induced neuroplasticity. In a study involving eighteen healthy participants, modulation of glutamatergic plasticity via dopaminergic agents showed distinct interactions: activation of D1-like dopamine receptors amplified glutamatergic NMDA receptor-mediated LTP-like plasticity induced by anodal stimulation, while concurrently abolishing the LTD-like plasticity typically observed with cathodal stimulation (Ghanavati et al., 2025). Conversely, general dopaminergic activation and specific D2-like receptor stimulation appeared to diminish glutamatergic NMDA receptor-mediated LTP-like effects while facilitating LTD-like plasticity, indicating a complex relationship between dopamine receptor activity and NMDA receptor-mediated glutamatergic signalling (Ghanavati et al., 2025). Collectively, these studies highlight the importance of the NMDA receptor and the broader glutamatergic system as essential mediators of the enduring synaptic plasticity induced by tDCS.

##### 2.2.1.2 GABAergic system

Recent evidence also indicates that tDCS can modulate neuronal excitability through interactions with the GABAergic system. Most research supports the view that anodal tDCS decreases GABA concentration or weakens GABAergic neurotransmission in the stimulated cortical region, thereby facilitating LTP-like neuroplastic effects (Antonenko et al., 2017; Heise et al., 2022; Patel et al., 2019; Stagg et al., 2014; Stagg et al., 2011; Tremblay et al., 2016). Nonetheless, findings have been inconsistent, with some studies reporting no significant changes in GABA concentration or GABA receptor activity following anodal tDCS (Nwaroh et al., 2020). In cats, Zhao et al. (2020) demonstrated a significant reduction in GABA concentration after anodal tDCS compared to sham stimulation, whereas cathodal tDCS primarily affected glutamatergic rather than GABAergic neurotransmission. A recent study by Ghanavati et al. (2025) supports the importance of both glutamatergic and GABAergic interactions in modulating cortical plasticity, highlighting that dopamine receptor subtypes (D1 and D2) significantly influence both GABA and glutamatergic neurotransmitter systems. This study suggests that dopaminergic modulation, via interactions with glutamatergic NMDA and GABAergic receptors, underlies the complex plasticity effects observed during tDCS, reinforcing the role of neurotransmitter-specific modulation as crucial to the variable outcomes reported across different tDCS protocols (Monte-Silva et al., 2010).

Together, these findings demonstrate that plasticity induced by tDCS is shaped by a delicate and dynamic balance between excitatory and inhibitory neurotransmission. Yet, while the glutamatergic and GABAergic systems form the neurochemical foundation of synaptic modulation, they do not fully explain the spectrum of plastic responses observed with tDCS. To capture the full complexity of tDCS-induced neuroplasticity, we must consider broader systems-level processes that extend beyond synaptic transmission. This includes the influence of additional neuromodulatory systems, as well as non-neuronal mechanisms such as glial function and cellular structural changes. The following sections explore these wider modulators of plasticity, beginning with the role of non-glutamatergic neurotransmitter systems and continuing through to cellular-level adaptations like proliferation and morphological remodelling.

#### 2.2.2 Modulators of plasticity

##### 2.2.2.1 Neurotransmitter systems

Beyond the primary excitatory and inhibitory systems, several additional neurotransmitter systems—including dopaminergic, cholinergic, serotonergic, and noradrenergic pathways—play modulatory roles in shaping neuroplasticity and may influence how tDCS-induced effects manifest. For instance, blockade of dopamine receptors has been shown to reliably abolish tDCS-induced plasticity, underscoring dopamine’s permissive role in facilitating plastic change in the motor cortex (Monte-Silva et al., 2013; Nitsche et al., 2009; Nitsche et al., 2006). However, this relationship is not linear: low and high doses of levodopa can suppress plasticity, while intermediate doses may enhance it (Monte-Silva et al., 2010). Similarly, cholinergic modulation appears to bias synaptic change towards potentiation, with receptor activation promoting LTP-like effects and antagonism blocking them (Auerbach and Segal, 1996; Blitzer et al., 1991; Hasselmo and Barkai, 1995). Adrenergic involvement is also evident; for example, monoamine reuptake inhibitors prolong the after-effects of anodal tDCS, whereas *β*-adrenergic receptor antagonism dampens both LTP- and LTD-like responses (Nitsche et al., 2004b). Importantly, these neuromodulatory systems are not universally required for tDCS-induced plasticity to occur. Rather, they influence the threshold, magnitude, and direction of plastic change in a context-dependent and often non-linear fashion (Nitsche et al., 2012). This suggests that tDCS-induced plasticity is contingent upon the state of these systems, but not necessarily dependent on their engagement. Their fluctuating activity levels, receptor distribution, and downstream signalling pathways can introduce considerable variability in outcomes, adding yet another layer of complexity to interpreting and predicting the effects of tDCS.

##### 2.2.2.2 Cell proliferation

In addition to synaptic and neuromodulatory effects, tDCS may also be capable of inducing non-synaptic plasticity, particularly at the level of cellular development and regeneration. A growing body of evidence suggests that electrical stimulation influences cell proliferation, neurite outgrowth, and morphological remodelling. Endogenous electric fields are known to play significant roles in neural development and repair processes (McCaig et al., 2005), and tDCS may similarly harness these mechanisms. For instance, Rueger et al. (2012) demonstrated that 10 consecutive days of cathodal tDCS applied to the rat primary motor cortex increased the number of proliferating cells and neural stem cells in the stimulated region by 160%. Complementary *in vitro* findings have shown that direct current electric fields can rapidly promote neurite extension (Koppes et al., 2011; Wood and Willits, 2009), modulate their orientation, and influence axonal guidance. Depending on the cell type, developmental stage, or experimental context, neurites have been observed to orient towards the cathode (Wood and Willits, 2009; Erskine and McCaig, 1995; Patel and Poo, 1984; Rajnicek et al., 2006), the anode (Cork et al., 1994), or perpendicular to the electric field (Pan and Borgens, 2010). *In vivo*, prolonged tDCS following ischemic injury in rats has been associated with increased dendritic spine density in surviving neurons at the infarct site, accompanied by improvements in motor function (Jiang et al., 2012). These findings suggest that extended tDCS protocols may support neurorestorative processes by promoting cellular growth and regeneration. However, the behavioural significance of tDCS-induced cell proliferation remains unexplored. Whether these structural changes translate into functionally meaningful outcomes—particularly in human populations—remains an important avenue for future research. Future research needs to directly assess the regenerative capacity of tDCS on cell proliferation and quantify any restorative effects on altered behavioural outcomes.

#### 2.2.3 Variability of tDCS effects

The effects of tDCS are often only interpreted through its impact on neurophysiological excitability—as reported above, predominately indexed by changes in TMS-evoked MEPs, TMS–EEG potentials, and metabolite changes (e.g., glutamate, GABA). The foundational research cited above establishes polarity-dependent after-effects on corticospinal excitability, whereby anodal tDCS increases cortical activity and producing LTP-like changes, whereas cathodal tDCS decreases cortical activity and producing LTD-like changes. However, contemporary evidence suggests a multitude of moderators—biological (age, sex, disease state, baseline excitability) and technical (stimulation parameters)—that shape both the magnitude and the direction of behavioural and neurophysiological outcomes. Indeed. umbrella and domain-specific meta-analyses increasingly emphasise these moderators when interpreting small-to-moderate average effects and occasional null results, emphasising the need to look beyond simple dichotomous and neuron-centric approaches to understanding the effects of tDCS (Ciechanski et al., 2018; Schroeder et al., 2020).

##### 2.2.3.1 Age

Head and brain anatomy change with ageing, including cortical atrophy and cerebrospinal fluid expansion (Fjell et al., 2010), altering the induced electric field and thereby moderating excitability responses (Indahlastari et al., 2020). Computational modelling in large ageing cohorts shows systematic age-related differences in electric field strength and distribution under conventional montages, implying that a fixed current dose can deliver different intracranial doses across the lifespan (Indahlastari et al., 2020; Ma et al., 2024). Indeed, experimental data in older adults show that anodal tDCS can reduce motor cortex GABA and alter sensorimotor network connectivity—physiological substrates that track excitability modulation—but with effects that differ from young adults, again consistent with age as a moderator (Antonenko et al., 2017). Collectively, these results argue for age-aware dose control and montage selection whenever excitability is the primary endpoint.

##### 2.2.3.2 Sex

Beyond age-related variability in the effects of tDCS, there are also sex-related anatomical differences that have the capacity to shift electric field focality and magnitude and thus excitability outcomes (Rudroff et al., 2020). Indeed, modelling data in humans indicate that tDCS-induced electric fields are higher in female head models than male head models at the same stimulation site and dose, indicating gender-related differences. This difference in gender related electric field distribution appears to be related to observed gender differences in a wide range of behavioural outcomes. For example, Weller et al. (2023) report that, regardless of stimulation condition, performance gains in a cognitive training task between the sexes were higher in females compared to males. Sex differences are also evident in clinical populations. Recent evidence suggests that sex modulates functional connectivity changes in primary progressive aphasia, with women receiving active tDCS showing greater language network connectivity changes compared to those in the sham condition (Licata et al., 2023). Beyond anatomy, endocrine state can shift baseline cortical physiology: menstrual-cycle–related hormone fluctuations are associated with changes in cortical excitability measures and manual dexterity in women (Zoghi et al., 2015). For excitability-focused studies, reporting sex, menstrual phase/contraceptive use, and—where possible—controlling for these factors must be considered.

##### 2.2.3.3 Disease state

The health of participants may also impact tDCS-induced effects, where many disorders and pathologies are characterised by altered baseline excitability and network physiology, which can shape tDCS after-effects (Chan and Han, 2020). For pathologies that impact the anatomy of the brain, this has a significant impact in electric fields and baseline excitability. For example, using current flow modelling in patients with stroke, van der Cruijsen et al. (2023) compared electric field variability when applying conventional anodal tDCS electrode configurations targeting the primary motor cortex in individualised MRI-based finite-element modelling in chronic stroke shows that a “standard” anodal M1 montage produces weaker, more variable, and sometimes polarity-inverted fields relative to healthy heads. These dose distortions mean that protocols validated in neurotypical samples often do not translate without individualised modelling and montage adjustment. Beyond anatomy, disease-specific network dynamics also impact tDCS-induced effects. An excitation–inhibition (E/I) imbalance is implicated across several neurodevelopmental conditions (Selten et al., 2018). In children with autism, active tDCS increased *α* power, narrowed α bandwidth, alongside gains on the Autism Behaviour Checklist and the Social Responsiveness Scale; with no comparable changes occurred after sham tDCS, consistent with a shift towards more balanced E/I dynamics (Kang et al., 2025). Taken together, pathology-specific anatomy and physiology should be treated as moderators of both dose (electric field strength) and response, and must be considered when interpreting cortical excitability endpoints and designing disease-appropriate stimulation parameters.

##### 2.2.3.4 Baseline excitability and state-dependence

The effects of tDCS may also be heavily impacted by baseline excitability and momentary brain state. For example, in the motor cortex, baseline excitability predicts the magnitude and sometimes the direction of plasticity after anodal or cathodal tDCS (Fresnoza et al., 2014). Applying tDCS during an active task directs current into the networks engaged by that task (e.g., frontoparietal or dorsal attention systems), leading to stronger and more specific plastic changes than stimulation at rest—demonstrating state dependence (Iordan et al., 2022). Similarly, when the default mode network (DMN) is dominant during internally focused thought, the current engages DMN-related regions rather than task-positive networks. In this context, stimulating DMN nodes alters the content of mind wandering—what people think about—without necessarily changing its frequency. This illustrates how ongoing cognitive state can redirect which networks are recruited by tDCS, reinforcing state dependence as a critical determinant of outcome. Together, baseline excitability and ongoing cognition govern which circuits are recruited and how durable plasticity will be. This highlights that controlling state and baseline excitability is crucial in determining tDCS effects.

##### 2.2.3.5 Stimulation parameters

tDCS variability is heavily shaped by how you stimulate: intensity, duration, polarity, and timing interact in non-linear ways, so the same montage can potentiate, null, or even reverse expected effects (Lewis et al., 2025a). For example, raising cathodal dose from 1 mA for 20 min to 2 mA for 20 min in the motor cortex can flip inhibition to facilitation, and prolonging anodal stimulation can likewise reverse LTP-like after-effects (Batsikadze et al., 2013). Montage choices then determine where and how current actually impacts the cortex: electrode placement of both the anode and cathode shapes the intracranial electric field through individual skull and CSF anatomy, while the electric field direction relative to cortical columns selects different corticospinal inputs (e.g., posterior–anterior vs. anterior–posterior), changing recruitment patterns and effect sizes (Opitz et al., 2015). Electrode size and arrangement add more variance: smaller, high-definition 4 × 1 arrays increase current density and focality compared with conventional pads (Kuo et al., 2013), but behavioural and physiological advantages over standard bipolar tDCS are mixed across tasks and samples (Mattavelli et al., 2025). Protocol timing also has an important role in tDCS outcome: pairing stimulation online with training versus applying it offline (Bikson and Rahman, 2013), and spacing repeated sessions to leverage metaplasticity, can amplify or mute outcomes (Besson et al., 2020; Lewis et al., 2025b). Therefore, tDCS dose is multidimensional, and accounting for how these variables interact is critical for interpreting and optimising its neurophysiological effects (Laakso et al., 2019).

Together, this section has discussed the immediate and enduring effects of tDCS on the neuronal components of the brain. Immediate changes in membrane potential and firing rate and timing cause larger, more enduring effects on the functional connectivity between brain regions and cortical excitability that can last for hours or days after stimulation cessation. These enduring effects and, indeed, the overall effects of stimulation are moderated by neuromodulatory systems including GABA, glutamate, and dopamine, and are differentially impacted by factors including age, sex, disease state, as well as stimulation parameters. Importantly, the effects of tDCS on neurons are only one layer of how electricity can impact the brain. Several of the moderators outlined above also act through non-neuronal targets—including astrocyte-mediated glutamatergic–GABAergic homeostasis, neurovascular coupling, and immune signalling—which can, in turn, modulate cortical excitability. The next section examines these non-neuronal effects of tDCS.

## 3 The non-neuronal mechanisms underlying tDCS

While the majority of tDCS research has focused on neuronal targets, there is growing recognition that tDCS-induced electric fields also influence non-neuronal elements within the brain, potentially contributing to the broader outcomes of neuromodulation. As our understanding of brain plasticity advances, it is increasingly important to account for the integrated function of the entire neurovascular unit—including neurons, glia, vasculature, and supporting cells—rather than isolating effects to neuronal activity alone. Building on the mechanisms described in the previous sections, following sections focus on how tDCS interacts with non-neuronal components. Specifically, we explore two broad domains of influence: the vascular structures that support cerebral blood flow and metabolic exchange, and the non-vascular elements that include microglia, astrocytes and oligodendrocytes.

### 3.1 The vascular mechanisms underlying tDCS

Given the tight link between neural firing and perfusion, early work treated any tDCS-related haemodynamic change as a downstream consequence of altered neuronal demand. Recent data, however, paint a more complex picture. Across preparation and imaging modality, polarity- and dose-dependent shifts have been recorded in blood vessels of every size—from pial arteries to endothelial tight-junctions in the BBB—suggesting that the vasculature itself is an active target of stimulation, not merely a passive consequence of it (Khadka and Bikson, 2022; Phillips et al., 2015; Shin et al., 2020). Building on the work of Bahr-Hosseini and Bikson (2021), we add the most recent findings on the effects of tDCS on the vasculature and extend the review to the extracellular space: anodal currents have now been shown to enlarge extracellular space and accelerate the glymphatic interchange of cerebrospinal and interstitial fluids (Wang and Monai, 2024).

#### 3.1.1 tDCS-induced effects on brain vasculature

Animal and human studies converge on the finding that tDCS produces polarity- and dose-dependent effects across the entire vascular tree. In rats, direct current pulses (10–20 V, 5–10 Hz) delivered to the exposed dura induce dilation of the middle meningeal artery in an intensity- and frequency-sensitive manner. Because this vasodilatory response persists even when the dura is physically separated from the underlying cortex, perivascular nerves rather than cortical neuronal activity appear to mediate the effect (Gozalov et al., 2008; Petersen et al., 2004; Kurosawa et al., 1995). Preclinical work further demonstrates similar dose-dependent changes in vascular diameter within larger cerebral arteries such as those in the circle of Willis (Emerson and Segal, 2001; Harder and Madden, 1987). Human experiments align with these observations: anodal tDCS reliably causes skin reddening and warmth due to superficial vessel dilation (Brunoni et al., 2011b; Durand et al., 2002) and modulates vasomotor reactivity of the middle cerebral artery, with montage-dependent, bilateral effects (List et al., 2015; Vernieri et al., 2004).

The microvasculature also exhibits direct susceptibility to stimulation. For instance, 10 min of low-intensity (0.1–1.5 mA) anodal stimulation applied across cultured brain endothelial monolayers increased permeability via electro-osmotic mechanisms, independently of neuronal activation (Cancel et al., 2018). Complementary *in vivo* studies show that 20 min sessions of 0.1–1 mA anodal tDCS transiently enhance BBB permeability to small (Na-fluorescein) and large (Dextran-70 kDa) solutes in rats (Shin et al., 2020). Multiphoton microscopy further reveals that tDCS accelerates solute diffusion throughout the extracellular matrix (Xia et al., 2020). However, in healthy humans, a single 20 min session of 2 mA anodal stimulation over the primary motor cortex did not alter salivary levels of S100B, a marker of BBB integrity, although subsequent exhaustive exercise did raise S100B concentrations. This suggests that a single session at typical therapeutic intensities is unlikely to significantly disrupt a healthy human BBB (Lewis et al., 2025c). Collectively, these findings strongly support the notion that cerebrovascular structures represent direct targets of tDCS, rather than merely reflecting secondary changes driven by neuronal activation. Future research needs to continue to quantify tDCS-induced changes in the BBB in humans using larger samples and more sensitive markers for BBB quantification.

#### 3.1.2 tDCS effects on cerebrospinal-interstitial fluid flux

Beyond the vasculature itself, low-intensity anodal stimulation may have an impact on the brain’s glymphatic system, the peri-arterial pathway that moves cerebrospinal fluid (CSF) into the interstitial space (ISF) to wash away metabolic waste (Mestre et al., 2020). This bulk flow is largely driven by aquaporin-4 (AQP4) water channels concentrated in the end-feet of perivascular astrocytes (Mestre et al., 2018). In mice, a single 10 min, 0.1 mA session enlarged the extracellular space, accelerated both CSF ingress and ISF efflux, and sped tracer wash-out through cervical lymph nodes (Wang and Monai, 2024). The effect hinged on astrocytic Ca^2+^ signalling and coincided with a transient increase in delta-band EEG power, yet AQP4 abundance was unchanged within the first 30 min (Wang and Monai, 2024). By contrast, when the same tDCS polarity was delivered for 30 min daily over 2 months in 3 × Tg-AD mice, AQP4 expression rose persistently and glymphatic tracer transit quickened (Luo et al., 2022). Together, these studies suggest that anodal currents can acutely hasten both CSF influx and solute-laden ISF efflux via astrocytic signalling, and—when applied repeatedly—induce structural AQP4 changes that maintain enhanced clearance. Whether a comparable glymphatic acceleration occurs in humans remains untested, but the rodent data place fluid-exchange pathways, alongside blood vessels, as polarity-sensitive targets that may underscore part of the therapeutic profile of tDCS. Future research is needed to better understand the role of glymphatic exchange and the removal of metabolic waste and its relationship to neuronal plasticity.

### 3.2 The non-vascular mechanisms underlying tDCS

In addition to its effects on brain vasculature, tDCS has been found to modulate other non-neuronal cells within the neurovascular unit, including astrocytes, microglia, and oligodendrocytes. Recent findings have indicated these cells provide their own essential role in neuronal functions, including plasticity, challenging the outdated notion of their passive role in neuronal function (Di Castro et al., 2011; Haydon and Carmignoto, 2006; Panatier et al., 2011) and neuromodulation.

#### 3.2.1 Astrocytes

Astrocytes are essential glial cells that regulate synaptic transmission and plasticity (Takata et al., 2011; Yang et al., 2003) by maintaining the extracellular environment (Verkhratsky et al., 2015), mediating energy substrate transfer from the bloodstream to neurons, and secreting bioactive molecules, thus contributing to the concept of a “tripartite synapse” (Perea et al., 2009). Given their role in LTP-like plasticity, tDCS-induced changes in astrocytic function may explain the effect of tDCS on synaptic plasticity. Recent findings suggest that anodal tDCS of 0.1 mA for 10 min results in cortex-wide, astrocytic derived Ca^2+^ elevations in mice (Monai et al., 2016). In addition, Cancel et al. (2022) investigated the effects of tDCS on the gene expression by astrocytes in mice. BDNF and FOS were chosen as markers of interest, as BDNF responsiveness relates to sensitivity to tDCS (Podda et al., 2016), and FOS is a reported marker of astrocytic activation (Rubio, 1997). The authors report FOS and BDNF gene upregulation in astrocytes in response to tDCS, providing further evidence of the role of astrocytes in tDCS-induced long-term effects. Using a different assessment of astrocytic activation, Callai et al. (2022) assessed astrocytic activation, also in mice, after tDCS via the quantification of S100B; an astrocytic derived protein. The authors report a significant increase in S100B in mouse CSF after 30 min of active tDCS, potentially indicating astrocytic activation. These findings suggest that the modulation of astrocytes may explain the long-term synaptic effects induced by tDCS. Drawing on similar mechanisms, future research should aim to better understand the relationship between astrocytic Ca+ signalling and its influence on glymphatic exchange and their combined influence on neuronal plasticity.

#### 3.2.2 Microglia

Microglia are a major glial cell element of the CNS and play a critical role as the resident macrophage in the CNS (Kreutzberg, 1996; Perry and Gordon, 1988). They serve as scavenger cells in the event of infection, inflammation, trauma, ischemia, and neurodegeneration in the CNS (González-Scarano and Baltuch, 1999; McGeer and McGeer, 1995). Research exploring the impact of tDCS on microglia has been driven by the opportunity to use tDCS to leverage the inflammatory response mediated by microglia. One study on rodents demonstrated that under both the anodal and cathodal electrodes, tDCS increased the density of microglia within the stimulated brain region, suggesting a shift of microglia towards their active state during tDCS (Rueger et al., 2012). When active, these microglial cells are implicated in immunomodulatory and neurogenesis effects (Pikhovych et al., 2016). Another study examining the effects of multi-session tDCS on microglia report that stimulation led to a polarity-dependent downregulation of the expression of Iba1, a marker of microglial activation (Pikhovych et al., 2016). Mishima et al. (2019) report that tDCS induces subtle, but significant, alterations in microglial motility in the cerebral cortex in awake mice. This was later confirmed and extended upon by Gellner et al. (2021), who report a wide spectrum of characteristic microglia features that are promoted by anodal DCS, including activation state dependent motility, to phagocytosis. Extending these findings to pathological contexts, Oishi et al. (2024) showed that direct current stimulation in mice with spinal cord injury facilitated motor function recovery by suppressing microglial hyperactivity in the damaged spinal cord. Similarly, Cherchi et al. (2022) demonstrated that cathodal tDCS improved outcomes following cerebral ischemia, with motor recovery associated with reduced microglial density, a shift towards more ramified and complex morphologies, and a corresponding attenuation of phagocytic, pro-inflammatory activity in perilesional regions. Together, these findings underscore that microglia are not only dynamically modulated by tDCS under physiological conditions but are also recruited in response to CNS damage, where their activity appears to mediate functional recovery.

In summary, current evidence establishes that tDCS can influence microglial density, motility, and morphology, with effects that vary according to polarity and experimental context. While studies in both healthy and damaged tissue converge on the capacity of tDCS to modulate microglial activation states, the precise mechanisms governing these responses, and their downstream contribution to neuroprotection or maladaptive inflammation, remain uncertain. Future research should prioritise delineating polarity- and region-specific effects of tDCS on microglia, clarifying whether these changes represent causal drivers of behavioural recovery, and systematically testing their relevance in translational models of CNS injury and disease.

#### 3.2.3 Oligodendrocytes

Oligodendrocytes play a vital role in myelinating axons in the CNS, enabling rapid electrical transmission through saltatory conduction of action potentials (Simons and Nave, 2016). The process of myelination and myelin repair relies in part on oligodendrogenesis, involving the migration, maturation, and differentiation of oligodendrocyte precursor cells into mature oligodendrocytes (Bradl and Lassmann, 2010). Currently, there is limited information regarding the effects of tDCS on oligodendrocytes, myelination, and remyelination of diseased cells. One *in vivo* study in rodents found an increase in oligodendrocyte-specific progenitor cells in the pyramidal tract after multiple days of tDCS stimulation, suggesting a potential avenue for investigating tDCS effects on oligodendrocyte production (Li et al., 2010). A single study has explored the potential of tDCS to enhance remyelination. In a mouse model using cuprizone-induced demyelination, researchers observed that tDCS treatment resulted in a greater degree of remyelination compared to the control group (Mojaverrostami et al., 2022). Furthermore, the combination of tDCS with mesenchymal stem cell transplantation further enhanced remyelination. Collectively, these findings indicate that tDCS may promote oligodendrocyte activation and remyelination processes, though the evidence remains preliminary. While existing evidence then appears to indicate oligodendrocyte activation following tDCS, additional research, including human trials, is needed. Future research in animal models that investigate the influence of tDCS-induced remyelination and its clinical utility for neurodegenerative diseases should be conducted before we can explore potential consequences for human behaviour.

In summary, available studies establish that tDCS can increase oligodendrocyte precursor cell proliferation and, under demyelinating conditions, facilitate remyelination, particularly when combined with adjunctive therapies. However, the evidence is sparse, limited to animal models, and the underlying mechanisms—whether through direct modulation of oligodendrocyte precursor cell differentiation, altered neuroinflammation, or secondary effects on neuronal activity—remain unresolved. Future research should aim to clarify the cellular pathways through which tDCS influences oligodendrogenesis and remyelination, and to determine whether these effects can be replicated in humans and leveraged for therapeutic benefit in demyelinating and neurodegenerative diseases.

#### 3.2.4 Immuno-metabolic responses to tDCS

Recent transcriptomic, proteomic, and metabolomic studies have begun to clarify how low-intensity tDCS initiates coordinated molecular adaptations extending far beyond acute membrane polarisation (Lee et al., 2022). Early RNA sequencing experiments in healthy rats demonstrated that even a single anodal session, depending on intensity, altered the expression of approximately one thousand cortical transcripts, notably influencing genes involved in inflammation, calcium-binding, and multiple neurotransmitter-receptor signalling pathways (Holmes et al., 2016). Chronic applications of tDCS further refine this picture, revealing a distinct neurotrophic profile linked to enhanced neurogenesis: genes promoting growth and survival (SOS, Raf, PI3K, Rac1, IRAK, and Bax) are consistently upregulated, while negative regulators of these processes (CHK, Crk, Rap1, p38, Ras, and NF-κB) are downregulated (Lee et al., 2022). Complementary metabolomic analyses have shown that tDCS influences metabolic pathways critical to mitochondria and bioenergetics, notably glycolysis and the tricarboxylic acid (TCA) cycle, speculated via calcium signalling pathways (Agrawal et al., 2024).

Immune genes display polarity-specific and time-sensitive responses. Within hours, anodal stimulation applied to rat brain upregulates major histocompatibility complex-I (MHC-I) transcripts, potentially enhancing neuronal tagging for microglial surveillance, thus limiting excessive sprouting. Conversely, cathodal currents elevate osteopontin, a protein associated with microglial polarisation towards a neuroprotective phenotype and promoting stem-cell proliferation (Rabenstein et al., 2019). Such polarity-dependent immune modulation is reflected functionally in disease models. For example, Liu et al. (2025) demonstrated that repeated anodal tDCS treatments shifted pro-inflammatory M1 microglia and neurotoxic A1 astrocytes towards anti-inflammatory M2 and protective A2 phenotypes, respectively. This shift suppressed the NF-κB → NLRP3 → IL-18 signalling axis, alleviating chronic pain symptoms in a rat osteoarthritis model. Proteomic analyses further enrich these transcriptomic findings by highlighting molecular changes directly at the synaptic level. Jung et al. (2019) reported that anodal tDCS administered prior to memory acquisition improved long-term memory performance and significantly altered the abundance of 184 hippocampal synaptoneurosome proteins, particularly those linked to glutamate receptors, ion-channel dynamics, and long-term potentiation scaffolds such as SHANK, GRIN, and GRIA. Extending these insights into disease models, Magri et al. (2021) found that tDCS selectively remodelled the blood transcriptome in 3 × Tg-AD mice but not in wild-type animals, suggesting potential peripheral biomarkers to track therapeutic responses in neurodegenerative conditions.

These studies highlight tDCS as a potent, context-dependent modulator of immunometabolic gene expression. By modulating neurogenic and metabolic gene networks, tDCS induces neuronal growth, plasticity, and improved energetic efficiency. Its ability to modulate immune pathways can either heighten microglial surveillance or establish a reparative microenvironment, contingent upon polarity and dose. At the synapse, tDCS adjusts protein expression to support sustained plasticity and memory formation. However, it must be noted that much of the research on immune-metabolic responses to tDCS have been conducted in vivo. Therefore, future research should extend beyond animal models and continue to use transcriptomics and proteomics in humans to better understand the genetic adaptations that occur after acute and chronic applications of tDCS. This is crucial for developing targeted stimulation protocols that maximise therapeutic plasticity while avoiding maladaptive immune or metabolic responses.

## 4 Clinical implications

The evidence that tDCS impacts the whole neurovascular unit has direct implications for approaches to how stimulation should be applied in clinical settings. One immediate implication relates to electrode placement. Current modelling studies and experimental data both highlight that electric fields are not uniformly distributed but instead interact with elements of the neurovascular unit, including astrocytes and capillary beds (Khadka and Bikson, 2022; Cancel et al., 2022). Placement strategies that account for these components of the neurovascular unit—beyond conventional EEG 10–20 coordinates—may yield more predictable modulation of target pathways, or prove to be more effective targets for therapeutic outcomes. For instance, positioning electrodes relative to denser vascular networks or ischemic areas could influence local changes in blood flow, BBB permeability, or extracellular ion clearance, thereby amplifying or constraining a broad range of behavioural outcomes.

A second consideration is polarity selection. While anodal stimulation has traditionally been associated with excitatory effects and cathodal with inhibitory effects, the evidence reviewed here indicates that polarity also governs non-neuronal processes. Anodal tDCS has been shown to promote BDNF expression, Ca^2+^-dependent signalling cascades, and oligodendrocyte progenitor differentiation (Monai et al., 2016; Mojaverrostami et al., 2022), processes potentially beneficial in pathological state recovery and remyelination contexts. Conversely, cathodal stimulation may attenuate hyperexcitability and reduce pro-inflammatory responses by modulating microglial motility and astrocytic activity (Monai et al., 2016). These polarity-specific pathways suggest that beyond traditional approaches to increase or decrease cortical excitability, researchers may also be able to shape molecular and cellular environments in line with therapeutic objectives.

Finally, insights into the broader patient phenotype underscore the importance of individualised approaches. Patients with altered BBB integrity, chronic inflammation, or demyelinating conditions may show distinct responsiveness to stimulation (Rossi et al., 2024; Shen et al., 2024). For example, tDCS-induced increases in BBB permeability could pose additional risks in acute injury contexts but may conversely facilitate drug delivery in carefully controlled scenarios (Petrovskaya et al., 2023). Similarly, in individuals with compromised vascular health, tDCS-evoked modulation of arterioles and capillaries may interact with baseline perfusion status in ways that alter efficacy (Peruzzotti-Jametti et al., 2013). These findings suggest that incorporating a broader understanding of the neurovascular unit profile should guide candidate selection and stimulation parameters to achieve more appropriate and effective therapeutic outcomes.

Taken together, these clinical implications show that translation of mechanistic findings requires moving beyond a neuron-centric model. Because tDCS acts on the neurovascular unit as a whole (see Table 1 for summary), choices regarding optimising clinical outcomes including electrode placement, polarity, and patient selection should be guided by cellular and systems-level interactions. Accounting for these dynamics offers a route to more targeted and effective therapeutic outcomes.

**Table 1:** Summary of the effects of direct current stimulation on elements of the neurovascular unit.

Neurovascular unit component	Duration of effects	Effect of tDCS
Neurons	Immediate	Linearly changes the polarisation of the membrane. Linearly changes the spike timing and firing rate of the neuron. Shift oscillatory dynamics of whole brain.
	Enduring	Induces LTD- and LTP-like synaptic plasticity. Induces cell proliferation, neurite outgrowth, and morphological remodelling.
Vasculature	Immediate	Dose-dependent changes in vascular diameter across pial arteries, arterioles, and capillaries. Dose-dependent change in blood brain barrier permeability. Dose-dependent increase in size of the extracellular space, causing accelerated cerebrospinal fluid ingress and efflux
Astrocytes	Immediate	Promotes Ca^2+^ signalling.
	Enduring	Increases FOS and BDNF gene expression.
Microglia	Immediate	Increases the density of microglia in the stimulated brain region. Creates changes in microglial motility.
	Enduring	Increases the recruitment of activated microglial cells in response to CNS damage.
Oligodendrocytes	Acute	Increases oligodendrocyte precursor cell proliferation. Facilitates remyelination under demyelinating conditions
Immuno-metabolism	Enduring	Alters genes linked to inflammation, calcium, and neurotransmitter signalling. Promotes growth and survival pathways, while reducing pro-inflammatory ones. Modifies metabolism (glycolysis, TCA cycle) and synaptic proteins, supporting neuroplasticity, memory, and disease resilience.

## 5 Conclusion

This narrative review has highlighted the diverse and complex neurophysiological effects of tDCS across multiple components of the neurovascular unit. While neuronal outcomes remain the most extensively studied aspect of tDCS physiology, findings of acute effects at both single-neuron and network levels remain notably inconsistent, likely due to variability in current flow, directionality, and methodological limitations. Although more consistent evidence exists regarding the long-term influences of tDCS, including effects on synaptic plasticity mechanisms such as LTP and LTD, the direct relationships between these physiological changes and meaningful behavioural or cognitive outcomes remain underexplored.

Importantly, emerging evidence indicates that tDCS effects extend beyond neurons alone, significantly modulating vascular function, BBB permeability, astrocytes, microglia, and oligodendrocytes. Recent studies employing transcriptomic, proteomic, and metabolomic techniques demonstrate that tDCS initiates molecular adaptations affecting inflammatory pathways, neurotrophic signalling, calcium-dependent processes, mitochondrial function, and synaptic proteins critical to long-term potentiation. The neurovascular unit thus represents an integrative and complex target of tDCS, suggesting that comprehensive modulation of these components collectively may better explain observed changes in cognitive, behavioural, and neuropsychological outcomes. Future research should move beyond a neuron-centric perspective to fully embrace a more integrative framework that considers interactions amongst all elements of the neurovascular unit. Such a holistic approach will enhance our understanding of how tDCS exerts its effects, thereby improving its therapeutic and cognitive-enhancement potential.
